# Bis(2-amino-4-phenyl-1,3-thia­zol-3-ium) tetra­chlorido­palladate(II)

**DOI:** 10.1107/S1600536814015360

**Published:** 2014-07-11

**Authors:** Reyna Reyes-Martínez, Rubén M. Carballo, Gonzalo J. Mena-Rejón, Simón Hernández-Ortega, David Cáceres-Castillo

**Affiliations:** a Facultad de Química, Universidad Autónoma de Yucatán, Calle 41 No. 421, Col. Industrial, CP97150, Mérida, Yucatán, Mexico; b Instituto de Química, Universidad Nacional Autónoma de México, Circuito exterior, Ciudad Universitaria, México, DF, 04510, Mexico

**Keywords:** crystal structure

## Abstract

The title compound, (C_9_H_9_N_2_S)_2_[PdCl_4_], consists of two monoprotonated 2-amino-4-phenyl-1,3-thia­zole molecules and one tetra­chlorido­palladate anion. The organic molecules exhibit a dihedral angle between the main rings planes of 31.82 (9)°. In the anion, the Pd^II^ atom is located on a crystallographic centre of symmetry with a square-planar geometry. In the crystal, the anions and cations are connected through bifurcated N—H⋯Cl hydrogen bonds, and these inter­actions lead to hydrogen-bonded tapes of cations and anions along [100].

## Related literature   

For the potential biological activity of compounds containing thia­zole rings, see: Annadurai *et al.* (2012 ▶); Alam *et al.* (2011 ▶). For the synthesis of thia­zole compounds, see: Cáceres-Castillo *et al.* (2012 ▶). For similar structures with protonated molecules, see: Form *et al.* (1974 ▶); Jin *et al.* (2011 ▶, 2013 ▶). For the crystal structure of non-protonated thia­zole, see: Au-Alvarez *et al.* (1999 ▶).
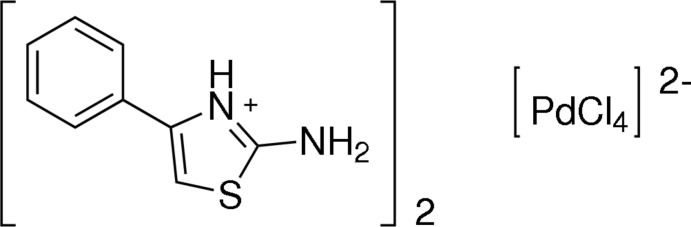


## Experimental   

### 

#### Crystal data  

(C_9_H_9_N_2_S)_2_[PdCl_4_]
*M* *_r_* = 602.68
Triclinic, 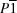

*a* = 7.2880 (2) Å
*b* = 8.9214 (3) Å
*c* = 9.8192 (3) Å
α = 66.258 (1)°
β = 73.778 (1)°
γ = 84.468 (1)°
*V* = 561.04 (3) Å^3^
*Z* = 1
Mo *K*α radiation
μ = 1.50 mm^−1^
*T* = 298 K
0.46 × 0.28 × 0.21 mm

#### Data collection  

Bruker APEXII CCD area-detector diffractometer
Absorption correction: analytical (*SADABS*; Bruker, 2012 ▶) *T*_min_ = 0.658, *T*_max_ = 0.842
4857 measured reflections
2060 independent reflections
1982 reflections with *I* > 2σ(*I*)
*R*_int_ = 0.026

#### Refinement  

*R*[*F* ^2^ > 2σ(*F* ^2^)] = 0.020
*wR*(*F* ^2^) = 0.051
*S* = 1.11
2060 reflections
143 parameters
3 restraints
H atoms treated by a mixture of independent and constrained refinement
Δρ_max_ = 0.26 e Å^−3^
Δρ_min_ = −0.30 e Å^−3^

### 

Data collection: *APEX2* (Bruker, 2012 ▶); cell refinement: *SAINT* (Bruker, 2012 ▶); data reduction: *SAINT*; program(s) used to solve structure: *SHELXTL* (Sheldrick, 2008 ▶); program(s) used to refine structure: *SHELXL97* (Sheldrick, 2008 ▶); molecular graphics: *ORTEP-3 for Windows* (Farrugia, 2012 ▶) and *DIAMOND* (Brandenburg, 2006 ▶); software used to prepare material for publication: *SHELXTL* and *PLATON* (Spek, 2009 ▶).

## Supplementary Material

Crystal structure: contains datablock(s) I. DOI: 10.1107/S1600536814015360/pj2013sup1.cif

Structure factors: contains datablock(s) I. DOI: 10.1107/S1600536814015360/pj2013Isup2.hkl

CCDC reference: 1011353

Additional supporting information:  crystallographic information; 3D view; checkCIF report

## Figures and Tables

**Table 1:** Hydrogen-bond geometry (Å, °)

*D*—H⋯*A*	*D*—H	H⋯*A*	*D*⋯*A*	*D*—H⋯*A*
N2—H2*B*⋯Cl2	0.89 (1)	2.41 (2)	3.237 (2)	155 (2)
N2—H2*A*⋯Cl2^i^	0.89 (1)	2.78 (2)	3.3572 (19)	123 (2)
N2—H2*A*⋯Cl1^ii^	0.89 (1)	2.44 (1)	3.291 (2)	159 (2)
N1—H1⋯Cl2	0.88 (1)	2.79 (2)	3.4028 (17)	129 (2)
N1—H1⋯Cl1	0.88 (1)	2.49 (2)	3.2593 (17)	147 (2)

Symmetry codes: (i) 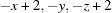; (ii) 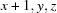.

**Fig. 1.**
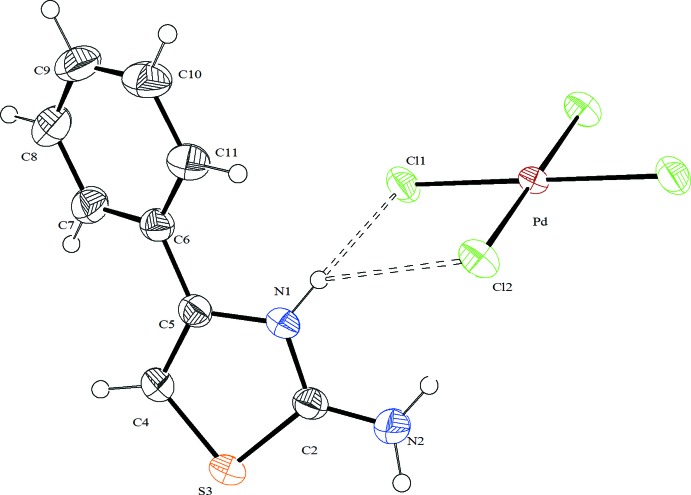
Molecular structure of title compound with displacement ellipsoids at the 40% probability. Hydrogen atoms are drawn as spheres of arbitrary radius.

**Fig. 2.**
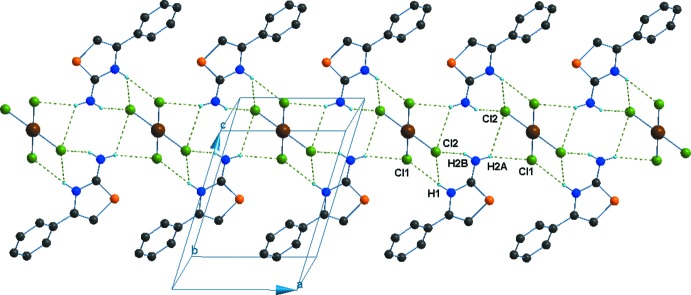
Linear arrangement due hydrogen bond patterns in crystal structure of the title compound.

## supplementary crystallographic information

### S1. Introduction

The thia­zole ring system is an important structural motif found in numerous molecules with potential biological activities, for instance; as anti­infective agents (Annadurai *et al.*, 2012; Alam *et al.*, 2011). On the other hand, in recent years there has been a growing inter­est in organic derivatives of transition metals in order to modify the biological properties of these organic compounds. Thus, in this opportunity we would like to report the crystal structure of bis-(2-amino-4-phenyl-1,3-thia­zolium) tetra­chloro­palladate (II).

### S2.1. Synthesis and crystallization

The compound 2-amino-4-phenyl-1,3-thia­zole was synthesized as reported by our group (Cáceres-Castillo *et al.*, 2012). The PdCl_2_ (25 mg, 0.14 mmol) was dissolved in 1 mL of concentrated HCl and then diluted with 5 mL of methanol. To the resulting mixture a methanol (5mL) solution of 2-amino-4-phenyl-1,3-thia­zole (50 mg, 0.28 mmol) was added. The reaction mixture was stirred for four hours at room temperature, after which time the resulting solution was allowed to slowly evaporate to produce brown X-ray diffraction quality crystals after few days.

### S2.2. Refinement

Crystal data, data collection and structure refinement details are summarized in Table 1.

All H atoms were included in calculated positions (C—H = 0.93 Å), and refined using a riding model with Uiso(H) = 1.2 Ueq of the carrier atom. H atoms on N were located in a Fourier map and refined isotropically with Uiso(H) = 1.2 × Ueq(N).

13 badly-fitted reflections were omitted from the final refinement.

### S3. Results and discussion

The title compound, [C_9_H_9_N_2_S]_2_[PdCl_4_], is centrosymmetric and consists of two monoprotonated 2-amino-4-phenyl-1,3-thia­zole molecules and one tetra­chloro­palladate anion. This compound, crystallized in the triclinic P-1 space group. The asymmetric unit is composed of one monoprotaned 2-amino-4-phenyl-1,3-thia­zole and half of the tetra­chloro­palladate anion, the other half is generated by application of an inversion centre. The dihedral angle between the planes of the phenyl and thia­zole rings in the cation is of 31.82 (9)°.This value is larger than those reported in other compounds containing the 2-amino-4-phenyl-1,3-thia­zole molecule, protonated (Form *et al.*, 1974; Jin *et al.*, 2013; Jin *et al.*, 2011) or in the free molecule (Au-Alvarez *et al.*, 1999). The angle C2—N1—C5 (115.25 (17)°) is similar in value other salts reported and is longer than that reported for the neutral compound (110.5 °).

The palladium atom of the anion is in a special position (0.5, 0, 1), *Wyckoff* site *1d*, and exhibits a square-planar geometry with Pd—Cl distances of 2.3031 (5) and 2.3061 (6) Å. The cation and the anion are linked by a bifurcate hydrogen bond between the chloride atoms and the hydrogen of the thia­zole ring (Figure 1). The NH and NH_2_ groups exhibit N—H···Cl hydrogen bonds with the chloride atoms generating a linear arrangement in the orientation [100] (Figure 2).

### Crystal data

**Table:**

(C_9_H_9_N_2_S)_2_[PdCl_4_]	*Z* = 1
*M**_r_* = 602.68	*F*(000) = 300
Triclinic, *P*1	*D*_x_ = 1.784 Mg m^−^^3^
*a* = 7.2880 (2) Å	Mo *K*α radiation, λ = 0.71073 Å
*b* = 8.9214 (3) Å	Cell parameters from 4506 reflections
*c* = 9.8192 (3) Å	θ = 2.4–25.4°
α = 66.258 (1)°	µ = 1.50 mm^−^^1^
β = 73.778 (1)°	*T* = 298 K
γ = 84.468 (1)°	Prism, brown
*V* = 561.04 (3) Å^3^	0.46 × 0.28 × 0.21 mm

### Data collection

**Table:**

Bruker APEXII CCD area-detector diffractometer	1982 reflections with *I* > 2σ(*I*)
Detector resolution: 0.83 pixels mm^-1^	*R*_int_ = 0.026
ω scans	θ_max_ = 25.4°, θ_min_ = 2.4°
Absorption correction: analytical (*SADABS*; Bruker, 2012)	*h* = −8→8
*T*_min_ = 0.658, *T*_max_ = 0.842	*k* = −10→10
4857 measured reflections	*l* = −11→11
2060 independent reflections	

### Refinement

**Table:**

Refinement on *F*^2^	Hydrogen site location: mixed
Least-squares matrix: full	H atoms treated by a mixture of independent and constrained refinement
*R*[*F*^2^ > 2σ(*F*^2^)] = 0.020	*w* = 1/[σ^2^(*F*_o_^2^) + (0.027*P*)^2^ + 0.0969*P*] where *P* = (*F*_o_^2^ + 2*F*_c_^2^)/3
*wR*(*F*^2^) = 0.051	(Δ/σ)_max_ < 0.001
*S* = 1.11	Δρ_max_ = 0.26 e Å^−^^3^
2060 reflections	Δρ_min_ = −0.30 e Å^−^^3^
143 parameters	Extinction correction: *SHELXL97* (Sheldrick, 2008), Fc^*^=kFc[1+0.001xFc^2^λ^3^/sin(2θ)]^-1/4^
3 restraints	Extinction coefficient: 0.015 (2)

### Special details

**Table:**

Geometry. All e.s.d.'s (except the e.s.d. in the dihedral angle between two l.s. planes) are estimated using the full covariance matrix. The cell e.s.d.'s are taken into account individually in the estimation of e.s.d.'s in distances, angles and torsion angles; correlations between e.s.d.'s in cell parameters are only used when they are defined by crystal symmetry. An approximate (isotropic) treatment of cell e.s.d.'s is used for estimating e.s.d.'s involving l.s. planes.

### Fractional atomic coordinates and isotropic or equivalent isotropic displacement parameters (Å^2^)

**Table:**

	*x*	*y*	*z*	*U*_iso_*/*U*_eq_	
Pd	0.5000	0.0000	1.0000	0.03103 (10)	
Cl1	0.54163 (7)	0.23160 (6)	0.77534 (6)	0.04971 (15)	
Cl2	0.78000 (7)	−0.10267 (6)	0.89335 (6)	0.04577 (14)	
N1	0.9774 (2)	0.2276 (2)	0.56952 (19)	0.0382 (4)	
H1	0.8651 (19)	0.185 (3)	0.628 (2)	0.046*	
C2	1.1103 (3)	0.2483 (2)	0.6301 (2)	0.0380 (4)	
N2	1.0983 (3)	0.1821 (2)	0.7791 (2)	0.0527 (5)	
H2A	1.203 (2)	0.195 (3)	0.804 (3)	0.063*	
H2B	1.001 (3)	0.112 (3)	0.837 (3)	0.063*	
S3	1.29130 (7)	0.37696 (7)	0.49056 (6)	0.04726 (15)	
C4	1.1739 (3)	0.4022 (3)	0.3520 (2)	0.0459 (5)	
H4	1.2196	0.4690	0.2481	0.055*	
C5	1.0096 (3)	0.3149 (2)	0.4107 (2)	0.0365 (4)	
C6	0.8732 (3)	0.3009 (2)	0.3309 (2)	0.0374 (4)	
C7	0.8497 (3)	0.4318 (3)	0.2005 (2)	0.0484 (5)	
H7	0.9175	0.5291	0.1660	0.058*	
C8	0.7259 (4)	0.4194 (3)	0.1206 (3)	0.0572 (6)	
H8	0.7096	0.5087	0.0340	0.069*	
C9	0.6278 (4)	0.2758 (3)	0.1694 (3)	0.0585 (6)	
H9	0.5474	0.2666	0.1143	0.070*	
C10	0.6482 (3)	0.1453 (3)	0.2995 (3)	0.0576 (6)	
H10	0.5800	0.0484	0.3328	0.069*	
C11	0.7696 (3)	0.1563 (3)	0.3820 (3)	0.0475 (5)	
H11	0.7815	0.0678	0.4707	0.057*	

### Atomic displacement parameters (Å^2^)

**Table:**

	*U*^11^	*U*^22^	*U*^33^	*U*^12^	*U*^13^	*U*^23^
Pd	0.02640 (13)	0.03439 (14)	0.03019 (13)	−0.00791 (8)	−0.00582 (8)	−0.00981 (9)
Cl1	0.0358 (3)	0.0484 (3)	0.0431 (3)	−0.0070 (2)	−0.0057 (2)	0.0025 (2)
Cl2	0.0345 (3)	0.0472 (3)	0.0495 (3)	−0.0033 (2)	−0.0015 (2)	−0.0183 (2)
N1	0.0298 (8)	0.0420 (9)	0.0380 (9)	−0.0078 (7)	−0.0058 (7)	−0.0112 (7)
C2	0.0326 (9)	0.0374 (10)	0.0416 (11)	−0.0027 (8)	−0.0092 (8)	−0.0129 (8)
N2	0.0494 (11)	0.0594 (12)	0.0434 (10)	−0.0154 (9)	−0.0169 (9)	−0.0074 (9)
S3	0.0326 (3)	0.0601 (3)	0.0447 (3)	−0.0143 (2)	−0.0069 (2)	−0.0151 (2)
C4	0.0387 (11)	0.0584 (13)	0.0365 (10)	−0.0118 (9)	−0.0051 (9)	−0.0147 (9)
C5	0.0321 (9)	0.0394 (10)	0.0376 (10)	−0.0008 (8)	−0.0062 (8)	−0.0164 (8)
C6	0.0329 (9)	0.0436 (10)	0.0393 (10)	0.0001 (8)	−0.0064 (8)	−0.0217 (9)
C7	0.0532 (13)	0.0504 (12)	0.0449 (12)	−0.0054 (10)	−0.0144 (10)	−0.0197 (10)
C8	0.0645 (15)	0.0669 (15)	0.0498 (13)	0.0047 (12)	−0.0254 (12)	−0.0265 (12)
C9	0.0519 (14)	0.0780 (17)	0.0680 (16)	0.0034 (12)	−0.0260 (12)	−0.0449 (14)
C10	0.0501 (13)	0.0593 (14)	0.0773 (17)	−0.0071 (11)	−0.0169 (12)	−0.0389 (13)
C11	0.0427 (11)	0.0453 (11)	0.0573 (13)	−0.0019 (9)	−0.0134 (10)	−0.0223 (10)

### Geometric parameters (Å, º)

**Table:**

Pd—Cl1	2.3031 (5)	C4—H4	0.9300
Pd—Cl1^i^	2.3031 (5)	C5—C6	1.468 (3)
Pd—Cl2^i^	2.3061 (5)	C6—C7	1.383 (3)
Pd—Cl2	2.3061 (5)	C6—C11	1.393 (3)
N1—C2	1.331 (2)	C7—C8	1.388 (3)
N1—C5	1.395 (3)	C7—H7	0.9300
N1—H1	0.876 (10)	C8—C9	1.369 (4)
C2—N2	1.319 (3)	C8—H8	0.9300
C2—S3	1.7179 (19)	C9—C10	1.373 (4)
N2—H2A	0.894 (10)	C9—H9	0.9300
N2—H2B	0.887 (10)	C10—C11	1.389 (3)
S3—C4	1.733 (2)	C10—H10	0.9300
C4—C5	1.343 (3)	C11—H11	0.9300
			
Cl1—Pd—Cl1^i^	180.0	C4—C5—C6	129.09 (18)
Cl1—Pd—Cl2^i^	90.134 (19)	N1—C5—C6	120.10 (17)
Cl1^i^—Pd—Cl2^i^	89.866 (19)	C7—C6—C11	119.06 (19)
Cl1—Pd—Cl2	89.866 (19)	C7—C6—C5	119.70 (18)
Cl1^i^—Pd—Cl2	90.134 (19)	C11—C6—C5	121.22 (19)
Cl2^i^—Pd—Cl2	180.00 (2)	C6—C7—C8	120.7 (2)
C2—N1—C5	115.25 (16)	C6—C7—H7	119.7
C2—N1—H1	120.8 (15)	C8—C7—H7	119.7
C5—N1—H1	121.9 (15)	C9—C8—C7	120.0 (2)
N2—C2—N1	123.49 (18)	C9—C8—H8	120.0
N2—C2—S3	125.28 (16)	C7—C8—H8	120.0
N1—C2—S3	111.19 (14)	C8—C9—C10	120.0 (2)
C2—N2—H2A	114.7 (17)	C8—C9—H9	120.0
C2—N2—H2B	115.3 (18)	C10—C9—H9	120.0
H2A—N2—H2B	128 (2)	C9—C10—C11	120.7 (2)
C2—S3—C4	90.03 (10)	C9—C10—H10	119.6
C5—C4—S3	112.71 (16)	C11—C10—H10	119.6
C5—C4—H4	123.6	C10—C11—C6	119.5 (2)
S3—C4—H4	123.6	C10—C11—H11	120.2
C4—C5—N1	110.80 (17)	C6—C11—H11	120.2

Symmetry code: (i) −*x*+1, −*y*, −*z*+2.

### Hydrogen-bond geometry (Å, º)

**Table:**

*D*—H···*A*	*D*—H	H···*A*	*D*···*A*	*D*—H···*A*
N2—H2*B*···Cl2	0.89 (1)	2.41 (2)	3.237 (2)	155 (2)
N2—H2*A*···Cl2^ii^	0.89 (1)	2.78 (2)	3.3572 (19)	123 (2)
N2—H2*A*···Cl1^iii^	0.89 (1)	2.44 (1)	3.291 (2)	159 (2)
N1—H1···Cl2	0.88 (1)	2.79 (2)	3.4028 (17)	129 (2)
N1—H1···Cl1	0.88 (1)	2.49 (2)	3.2593 (17)	147 (2)

Symmetry codes: (ii) −*x*+2, −*y*, −*z*+2; (iii) *x*+1, *y*, *z*.
